# INFERRING ADL BEHAVIORS FROM HIGH DENSITY, AMBIENT, INTERNET OF THINGS SENSOR DATA USING MACHINE LEARNING

**DOI:** 10.1093/geroni/igz038.2369

**Published:** 2019-11-08

**Authors:** Susann M Keohane, Scott N Gerard

**Affiliations:** 1 IBM, Austin, Texas, United States; 2 IBM, Durham, North Carolina, United States

## Abstract

To address the growing desire for older adults to age-in-place, there has been an influx of research and development in telehealth services that utilize smart home and ambient assisted living (AAL) technology. AAL technology is designed to care for, remotely monitor, and support healthy aging. The challenge with AAL technology is to accurately classify an older adults’ activities and provide a baseline for detecting changes in physical and cognitive state. We employ (1) rules-based, (2) Latent Dirichlet Allocation (LDA) and (3) auto-encoders (a type of neural network) to infer activities of daily living and instrumented activities of daily living behavior from low-level Internet of Things sensor data. We apply our techniques to a large dataset over 5 older adults in independent living apartments, for over 5 months with 30 to 50 ambient sensors per apartment. Findings highlighted three main themes: (1) lessons learned, (2) an evaluation of the various approaches employed, and (3) a semi-automated methodology to label large volumes of raw sensor data.

